# Digital Platform to Continuously Monitor Patients Using a Smartwatch: Preliminary Report

**DOI:** 10.2196/40468

**Published:** 2022-09-15

**Authors:** Kaio Jia Bin, Lucas Ramos De Pretto, Fabio Beltrame Sanchez, Linamara Rizzo Battistella

**Affiliations:** 1 Instituto de Medicina Física e Reabilitação do Hospital das Clínicas Faculdade de Medicina Universidade de São Paulo São Paulo Brazil

**Keywords:** smartwatch, digital health, telemedicine, wearable, telemonitoring, mobile health, digital platform, clinical intervention, sensitive data, clinical trial

## Abstract

**Background:**

Monitoring vital signs such as oximetry, blood pressure, and heart rate is important to follow the evolution of patients. Smartwatches are a revolution in medicine allowing the collection of such data in a continuous and organic way. However, it is still a challenge to make this information available to health care professionals to make decisions during clinical follow-up.

**Objective:**

This study aims to build a digital solution that displays vital sign data from smartwatches, collected remotely, continuously, reliably, and from multiple users, with trigger warnings when abnormal results are identified.

**Methods:**

This is a single-center prospective study following the guidelines “Evaluating digital health products” from the UK Health Security Agency. A digital platform with 3 different applications was created to capture and display data from the mobile phones of volunteers with smartwatches. We selected 80 volunteers who were followed for 24 weeks each, and the synchronization interval between the smartwatch and digital solution was recorded for each vital sign collected.

**Results:**

In 14 weeks of project progress, we managed to recruit 80 volunteers, with 68 already registered in the digital solution. More than 2.8 million records have already been collected, without system downtime. Less than 5% of continuous heart rate measurements (bpm) were synchronized within 2 hours. However, approximately 70% were synchronized in less than 24 hours, and 90% were synchronized in less than 119 hours.

**Conclusions:**

The digital solution is working properly in its role of displaying data collected from smartwatches. Vital sign values are being monitored by the research team as part of the monitoring of volunteers. Although the digital solution proved unsuitable for monitoring urgent events, it is more than suitable for use in outpatient clinical use. This digital solution, which is based on cloud technology, can be applied in the future for telemonitoring in regions lacking health care professionals. Accuracy and reliability studies still need to be performed at the end of the 24-week follow-up.

## Introduction

### Background

Since the World Health Organization declared the novel coronavirus a pandemic on March 11, 2020 [1], with more than 470 million cases of infection and more than 6 million deaths confirmed [2], digital transformation of health care worldwide has accelerated [3-5].

In this scenario where the most frequent comorbidities are hypertension (55%), coronary artery disease and stroke (32%), and diabetes (31%) [6], monitoring vital signs such as oximetry, blood pressure, and heart rate can be of paramount importance to monitor the evolution of patients infected by COVID-19.

Thus, wearable devices, such as smartwatches, are key actors in revolutionizing medicine through mobile health (mHealth) and eHealth, allowing continuous and longitudinal health monitoring outside of health care facilities [7].

Due to the ease of use of smartwatches—initially aimed at consumers concerned about their own health—several studies have shown interest in their application for remote monitoring [8] and as a tool for telemonitoring and early detection of respiratory symptoms [9,10], heart disease [11-14], and remote physical therapy [15].

Smartwatches can record clinical data in a way that feels organic and unobtrusive to the user, enabling the construction of a database that will facilitate, with the aid of artificial intelligence, the recognition of biomarkers capable of expanding the mechanisms of prediction, prevention, and health event intervention.

There are reports in the literature discussing smartwatch data collection [16], with the most recent already suggesting the possibility of early detection of COVID-19 [9,10] and atrial fibrillation [12-14]. However, for wearable devices to be really usable in a clinical setting, a digital platform that is easily accessible to health professionals is necessary.

### Motivation

Aiming to enable the future of telemonitoring, early detection, and remote therapies, there is a need for a digital web platform capable of collecting data from smartwatches continuously and effectively.

### Aim

This study aimed to build a digital solution that displays vital sign data from smartwatches, collected remotely, continuously, reliably, without the need on manual input, and from multiple users, triggering warnings when abnormal results are identified.

## Methods

The digital solution was developed to support an entire clinical study involving remote monitoring of patients. Its role is to guarantee anonymity, completeness, and reliability of the data collected, as well as to consolidate different sources of information input.

### Ethical Considerations

This project was submitted to the Ethics and Research Committee of the Hospital das Clinicas da Faculdade de Medicina of University of São Paulo (HCFMUSP; CAAE: 51711921.3.0000.0068 and Opinion number: 4,975,512).

### Study Type

This is a single-center prospective study following the guidelines “Evaluating digital health products” from the UK Health Security Agency [17], with local adaptations for the Brazilian population and project context, still in the design and test phases of a digital product.

A descriptive study, “Analysis of routinely collected data” [18], was conducted using data collected from the digital solution to a website platform by the team of executing researchers.

### Data Transfer

There are several ways to collect data from a smartwatch to a digital platform, as described by de Arriba-Pérez et al [19]. To ensure the validity and protection of the volunteer´s sensitive data [20], we developed an application to be used in parallel with the manufacturer’s app.

In this paper, only data used for clinical follow-up of volunteers were selected, namely blood pressure, oxygen saturation, heart rate, and sleep quality information (Table 1).

**Table 1:** Information collected from the smartwatch for this study.

Data	Collection	Present at digital solution
Steps	Automatically	No
Flights of stairs	Automatically	No
Exercise time	Automatically	No
Sleep quality	Automatically	Yes
Heart rate	Automatically	Yes
Oxygen saturation during sleep	Automatically	No
Oxygen saturation	User action	Yes
Blood pressure	User action	Yes
Weight	User action	No
Height	User action	No
Quantity and type of liquid ingested	User action	No
Quantity and type of food ingested	User action	No

### Data Flow

Vital sign data are captured by the smartwatch during its use by the volunteer. The smartwatch then synchronizes with the volunteer’s smartphone, and the data are transferred to the manufacturers’ health apps.

A custom application, called LIKA-App, reads the data from those health apps and transfers the data to a cloud server. A cloud data collector installed on the internal server of HCFMUSP downloads the data from the cloud server.

A second, complementary digital solution, LIKA-Web, makes the acquired data available for analysis and allows alerts to be set up through a user-friendly interface.

**Figure 1:**
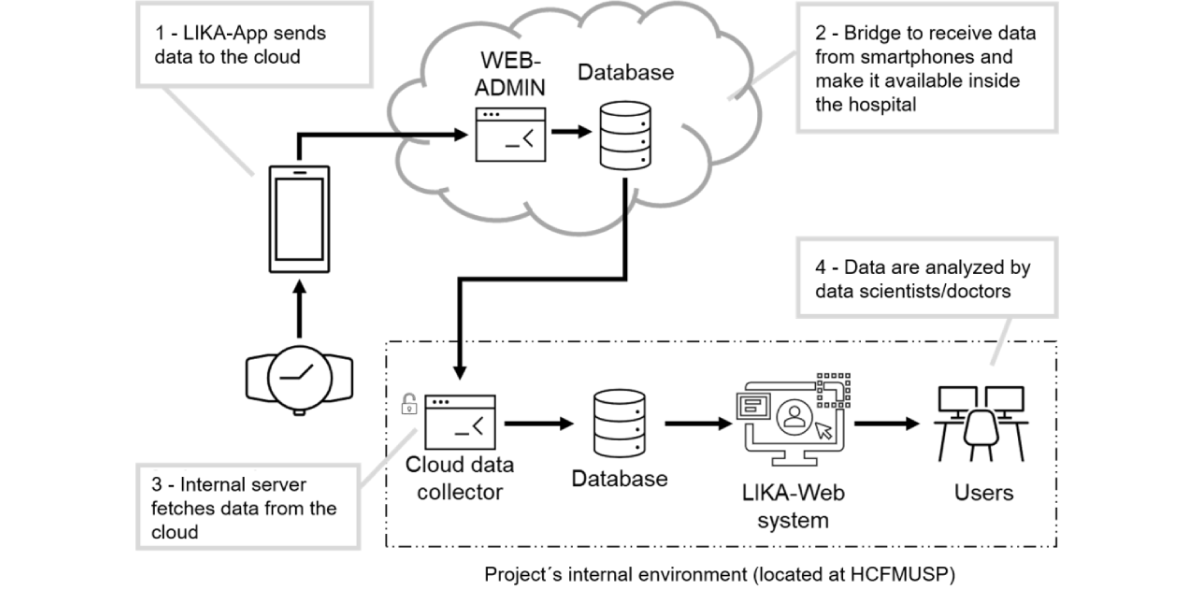
Data flow from smartwatches. HCFMUSP: Hospital das Clinicas da Faculdade de Medicina of University of São Paulo.

The data flow is shown in Figure 1.

**Figure 2:**
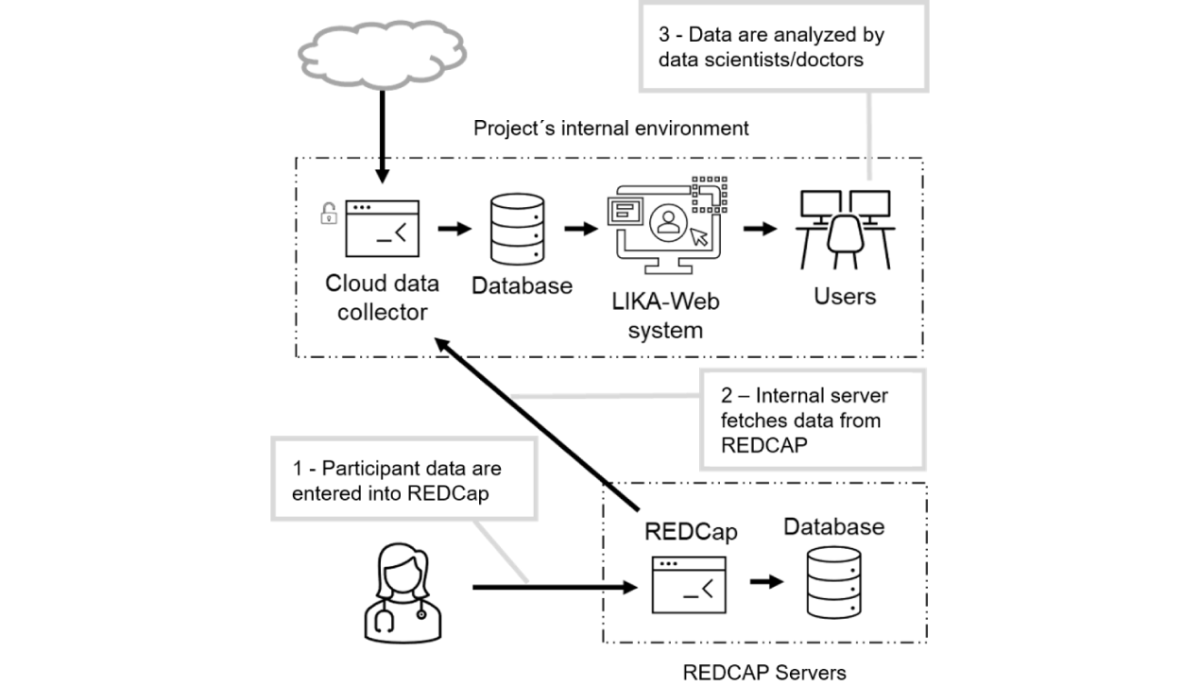
Data flow from Research Electronic Data Capture (REDCap).

In addition to those collected by the smartwatch, data obtained by gold standard devices will also be persisted in the project’s internal server database. These data will be entered manually in the Research Electronic Data Capture (REDCap) [21] system and later imported and incorporated into the study’s database. A diagram to visualize the integration between REDCap and the LIKA-Web solution can be found in Figure 2.

### Digital Solutions

#### LIKA-App

This app is a digital solution installed on volunteers’ smartphones that captures specific health information from the wearable device and synchronizes it with the study´s data cloud (WEB-ADMIN server). This application runs on Android smartphones and was developed and improved by the manufacturer to accommodate the requirements of this study.

#### WEB-ADMIN

This is the module responsible for receiving data obtained from the LIKA-App and storing it in the study’s data cloud for later use by the LIKA-Web. It also tracks the date and time of the latest synchronization of each participating volunteer’s smartwatch. This module provides the REST APIs [22] consumed by LIKA-App and a structured database (PostgreSQL).

#### LIKA-Web

LIKA-Web is the module responsible for supporting the remote monitoring project. It is a digital solution capable of retrieving information from different data sources for each volunteer. A user-friendly interface allows the team of researchers and clinicians to follow the day-to-day activities of the clinical study, as well as receiving alerts about vital signs and synchronization information.

To allow fast development and good reliability, LIKA-Web was built using Java Spring Boot framework (back-end) and Bootstrap/HTML/JQuery (front-end), with a MySQL database.

### Data Sync

The synchronization of data between the manufacturer’s health app and the LIKA-App takes place either under a deliberate request by the user, at any time, or automatically, once a day, but only while the app is active on the user’s phone. This synchronization is dependent on internet connectivity.

Once the LIKA-App retrieves information from the manufacturer’s application, these data are available to be synchronized with LIKA-Web, which takes place automatically via WEB-ADMIN every hour or under deliberate request by the Web Administrator at any time.

### Functionality

The platform offers the functionality described in the following paragraphs.

User registration grants users access to the LIKA-Web system.

During volunteer registration, for privacy protection, the volunteer’s name is not registered in our internal system (LIKA-Web). Instead, a unique user identifier is created and registered in the LIKA-App mobile app.

Volunteer risk alerts are triggered to warn the health care team based on the data received from the volunteers.

Data sync alerts identify volunteers who have not synced their smartwatch data in the last 72 hours.

The latest data log is an interface that logs the latest data obtained from each volunteer, along with the date and hour.

The data display interface displays the vital sign data obtained from the smartwatches.

### Wearable and Gold Standard Device

Each volunteer was provided with a smartwatch (Samsung Galaxy 4). The gold standard devices used in this study are a noninvasive blood pressure monitor, (G-TECH model GP400 [ANVISA registration nº 80275319016] with 2 AAA batteries) and a pulse oximeter (AFK YK009 [ANVISA registration 81995169005]) for noncontinuous monitoring.

A smartphone (Samsung A52) was provided to volunteers who did not have a Samsung mobile phone.

### Data Settings

Among the HCFMUSP collaborators, 80 volunteers were selected to use the smartwatch over 24 weeks, with daily visits by research monitors.

The following data were collected from the smartwatch by the LIKA-App: heart rate, oxygen saturation, blood pressure, sleep count, and sleep intensity.

Heart rate is configured to be collected continuously in 1-minute intervals. The smartwatch sends the value in bpm to the LIKA digital solution.

Oxygen saturation can be collected manually by the user or continuously during sleep.

Blood pressure is collected upon user request, and the smartwatch needs to be calibrated with a gold standard blood pressure device once every 28 days.

Sleep count is a feature that marks the beginning and end of the volunteer's sleep period. Sleep intensity data record the beginning and end of each sleep phase [23]. Both types of sleep data are automatically collected by the smartwatch without the need for user intervention.

For each of these values, the digital platform also collects the date and time of their record by the smartwatch, as well as the date and time when the digital platform synchronized and received the data.

Furthermore, every day, for 24 weeks, a research monitor meets with the volunteers and simultaneously collects oxygen saturation and blood pressure data from both the volunteer’s smartwatch and gold standard devices. Those values, along with date and beginning and end times (as displayed by the smartwatch), are registered on a paper form and later transcribed to REDCap, in order to analyze the reliability and agreement between the gold standard and smartwatch data.

For the purpose of this study, the automatic synchronization interval between LIKA-Web and LIKA-App is set to every 1 hour.

### Digital Platform Reliability Analysis

To determine the reliability of the data captured by the LIKA platform (as seen in Figure 1) at the end of the study, the data collected from the smartwatch and manually recorded in REDCap will be compared with those directly captured by WEB-ADMIN.

An active search will be carried out by combining data manually annotated in REDCap, by day, time, and volunteer, within the internal server database.

**Figure 3:**
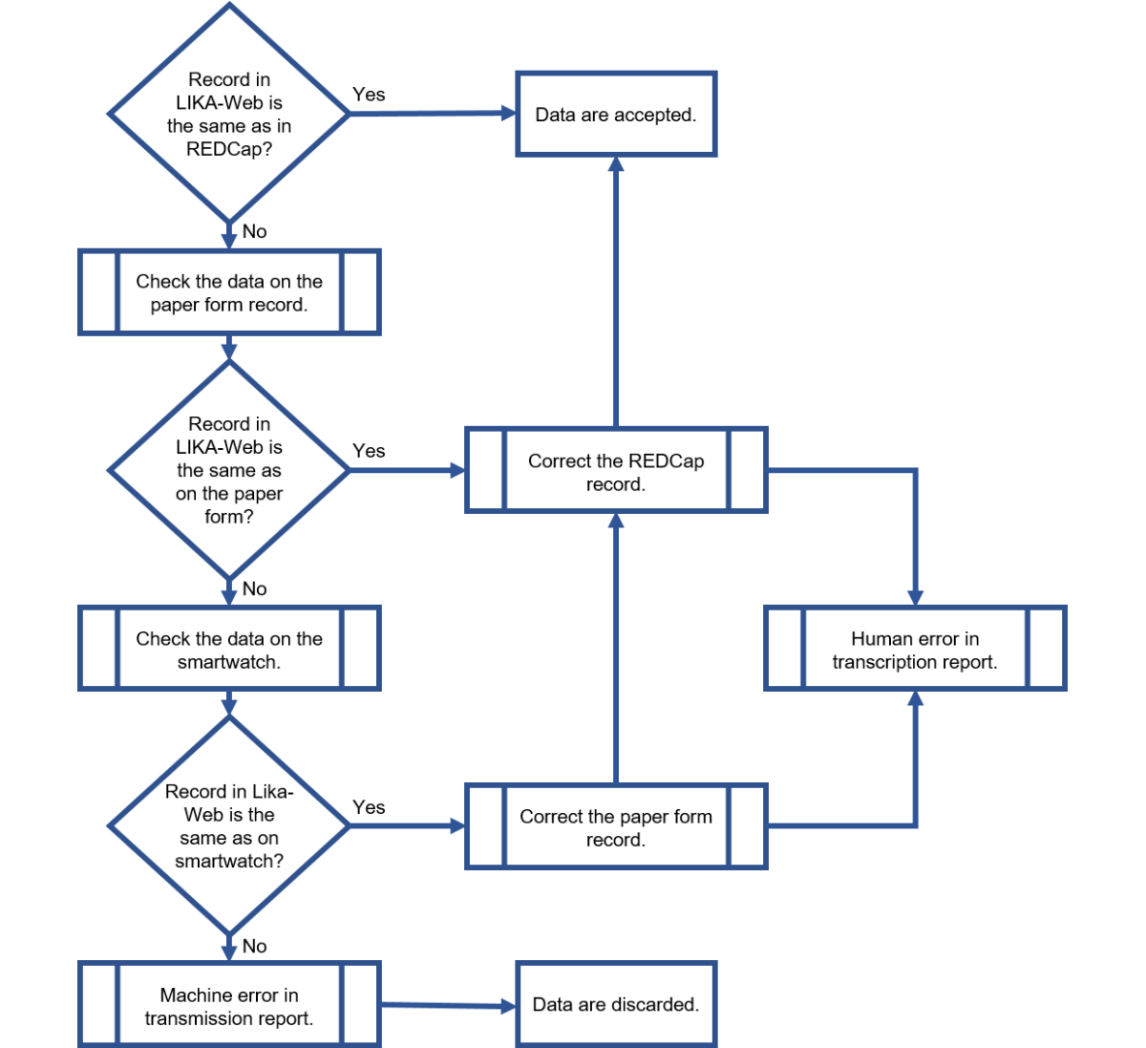
Comparison of the flow of data collected by the digital platform with those recorded in Research Electronic Data Capture (REDCap), on the paper form, and by smartwatch.

The comparison flow is in Figure 3.

### Alert System Measurement

The following triggers were defined for the blood pressure alerts, based on the manufacturer's recommendations [24]: systolic blood pressure ≤70 mm Hg or >180 mm Hg and diastolic blood pressure ≤40 mm Hg or >120 mm Hg.

Every time one of these events is triggered by the digital platform, the value, date, hour, and volunteer’s identifier are displayed on the dashboard.

With the intention of monitoring the use of the smartwatch by the volunteers, alerts regarding oxygen saturation and heartbeat per minute were also implemented on the digital platform.

Considering the normal oxygen levels in a pulse oximeter usually range from 95% to 100% and hypoxemia is an oxygen saturation of less than 90% [25], the digital platform was set up to alert when the value reaches below 88%.

Similarly, considering that the diagnosis of sinus bradycardia requires an electrocardiogram showing a normal sinus rhythm at a rate lower than 60 bpm [26], the digital platform was set up to alert when the heart rate falls below 40 bpm.

## Results

Running without interruption since February 25, 2022, a total of 68 volunteers were already being monitored by the LIKA-Web platform as of May 28, 2022 (Table 2), with 3 dropouts in the first week of the study.

Among the dropouts, a lack of time to attend the daily face-to-face data collection visits was the leading reason for abandoning the study. Data from these 3 dropout volunteers (465 records) were excluded from the analysis.

A total of 2,772,766 records were captured: 2,645,286 for continuous heart rate, 7423 for oxygen saturation, 4742 for blood pressure, 3599 for sleep count (marks the beginning and end of sleep), and 111,716 for sleep intensity (marks the beginning and end of each phase of sleep).

There were 9 users with continuous heart bpm data synchronization issues, 1 with oxygen saturation data issues, and 10 with blood pressure data synchronization issues (Table 3).

We noticed that many heart rate synchronization issues were due to the smartwatch battery management configuration: The smartwatches provided to the volunteers were configured in the continuous acquisition mode; however, as soon as the smartwatch entered battery saving mode, the configuration reverted back to only 1 measurement every 10 minutes. To address this problem, the research team advised volunteers not to use the battery saving mode on their devices.

Regarding the oxygen saturation issues, there was a synchronization problem with a single, newly recruited volunteer, and the research team was working to identify the reason.

Finally, for the blood pressure synchronization issues, we verified that the cause was a sharing permission between Samsung Monitor and Samsung Health App. The devices were configured with all permissions when the smartwatches were handed out to the volunteers; however, for unconfirmed reasons (likely system updates), the permission configurations were lost. To solve this problem, the research team reconfigured the data sharing permission as soon as the problem was identified by our data sync alert system.

Based on the collected data, descriptive statistics were performed across all the records obtained (Table 4). The overall results for heart rate and oxygen saturation show a distribution (between the 25th and 75th percentiles) within the expected normative values, indicating that there were no persistent abnormal readings from our volunteers.

**Table 2:** Total of users by weeks of the project.

Week number	New users, n	Number of users who dropped out, n	Total users, n
1	2	0	2
2	7	0	9
3	8	0	17
4	5	0	22
5	9	0	31
6	6	0	37
7	4	1	40
8	9	1	48
9	2	1	49
10	7	0	56
11	3	0	59
12	2	0	61
13	6	0	67
14	1	0	68

**Table 3:** Total data records by type and per user (N=2,772,766).

Data type	Records, n	Total users, n	Users with no synchronization, n
Continuous heart rate (bpm)	2,645,286	68	9
Oxygen saturation	7423	68	1
Blood pressure	4742	68	10
Sleep	3599	68	0
Sleep intensity	111,716	68	0

**Table 4:** Descriptive statistics of all the collected vital signs.

Data type		Results				
		Mean (SD)	95% CI	25th percentile	50th percentile	75th percentile
Heart rate (bpm)		78.29 (13.63)	78.27-78.30	69.00	77.00	86.00
Oxygen saturation (%)		94.37 (3.47)	94.29-94.45	93.00	95.00	97.00
**Blood pressure (mm Hg)**						
	Systolic	126.78 (15.06)	126.35-127.21	116.00	127.00	136.00
	Diastolic	82.45 (12.16)	82.10-82.79	73.00	83.00	92.00

Similarly, the blood pressure results present a distribution mostly inside the range expected for resting blood pressure. Individual averages of heart rate for each patient also present a normative distribution, with lower (Q1), median (Q2), and upper (Q3) quartile values of 74.57 bpm, 78.24 bpm, and 82.66 bpm, respectively. The distribution of the individual means for oxygen saturation is also compatible with the normative range but shifted to slightly lower values: 93.67%, 94.64%, and 95.24% (Q1, Q2, and Q3, respectively).

The same observations were made for individual averages for systolic (Q1: 116.69 mm Hg; Q2: 120.50 mm Hg; Q3: 129.06 mm Hg) and diastolic (Q1: 76.16 mm Hg; Q2: 82.16 mm Hg; Q3: 89.63 mm Hg) blood pressures.

Data regarding sleep intensity were also analyzed. The smartwatch records sleep intensity at 4 different levels: still awake, light sleep, deep sleep, and rapid eye movement (REM).

The total sleep time recorded was verified, and the percentage of this total spent at each sleep intensity was calculated. The results are shown in Table 5, where it can be seen that more than one-half (590,433/1,046,195, 56.43%) of the recorded sleep time was spent in light sleep, and only about 20% (213,716/1,046,195, 20.43%) was spent in REM sleep. Yet, despite this, we found that all volunteers managed to achieve REM sleep at least once.

Still, regardless of the distributions in Table 3, a few vital sign records fell outside the range of normative values, and, to monitor those, the alert system within our framework was used. In all, 421 alerts were generated (Table 6).

To assess whether the platform can be used by the clinical team as a digital health care solution, it is very important that the data be available in the digital platform as soon as possible after being recorded by the smartwatch.

The time interval between the moment of recording the continuous beats per minute (cBPM) and the moment the value is available on the digital platform depends on the synchronization between the manufacturer's health app and LIKA-Web, via the LIKA-App.

In total, 34 Samsung A52 smartphones were provided to volunteers who did not already have a Samsung mobile phone. In addition, 34 volunteers provided their own Samsung smartphone (multiple models) for use in this study. The proportions of cases with synchronization issues were similar, regardless of whether the volunteer was using their own smartphone (Table 7).

As shown in Table 8, less than 5% of records synced within the first 2 hours of interval time, either in the group with their own smartphone (55,955/1,349,706, 4.15%) or in the group with a smartphone provided by the study (64,943/1,295,580, 5.01%). On the other hand, most of the data were synchronized within 24 hours in both groups, and only less than 10% of data were synced after 7 days.

So far, no downtime events have been recorded on the LIKA-Web platform, and this project is scheduled to end on October 23, 2022.

**Table 5:** Time spent in each sleep stage across all recorded minutes (1,046,195 minutes).

Sleep phase	Overall recorded minutes (% of total)
Awake	122,898 (11.75)
Light sleep	590,433 (56.43)
Deep sleep	119,148 (11.39)
REM^a^ sleep	213,716 (20.43)

^a^REM: rapid eye movement.

**Table 6:** Type and number of alerts and number of volunteers who triggered each alert.

Type of alert	Number of alerts (n=421)	Number of volunteers (n=45)
Maximum diastolic blood pressure (≥180 mm Hg)	1	1
Minimum diastolic blood pressure (≤70 mm Hg)	0	0
Maximum systolic blood pressure (≥120 mm Hg)	3	1
Minimum systolic blood pressure (≤40 mm Hg)	0	0
Low oxygen saturation (<88%)	414	43
Low heart rate (<40 bpm)	3	2

**Table 7:** Number of volunteers with synchronization (sync) errors.

Volunteers	Sync OK (n=59), n	Sync error (n=9), n	cBPM^a^ records (n=2,645,286), n
Wth own smartphone	30	4	1,349,706
With study´s smartphone	29	5	1,295,580

^a^cBPM: continuous beats per minute.

**Table 8:** Synchronization time according to the smartphone used.

Sync time	Study’s smartphone (n=1,295,580)			Volunteer’s smartphone (n=1,349,706)	
	Number synced, n (%)	Accumulated %	Number synced, n (%)		Accumulated %
<1 hour	5051 (0.39)	0.39	4400 (0.33)		0.33
1-2 hours	59,892 (4.62)	5.01	51,555 (3.82)		4.15
3-6 hours	193,524 (14.94)	19.95	166,518 (12.34)		16.48
6-12 hours	278,549 (21.50)	41.45	266,397 (19.74)		36.22
13-24 hours	349,405 (26.97)	68.42	452,547 (33.53)		69.75
1-2 days	143,495 (11.08)	79.49	196,683 (14.57)		84.32
3-7 days	154,646 (11.94)	91.43	131,064 (9.71)		94.03
7-14 days	78,115 (6.03)	97.46	55,495 (4.11)		98.14
>14 days	32,903 (2.54)	100	25,047 (1.86)		100

## Discussion

### Principal Findings

The LIKA-Web platform has been stable and is ready to fulfill its role of receiving data from mobile devices.

In the 14 weeks of follow-up as of the writing of this manuscript, we had not had any interruption of its service, and it is being actively used to monitor the health of the volunteers as well as to verify if they performed the daily synchronization with the platform.

In the first week of recruitment, the equipment was delivered to the volunteers on the day of their consultation, which incurred a lot of difficulty with the setup time. It took more than 90 minutes just to charge the smartwatch, pair it with the mobile phone, and install the apps.

From the second week onwards, equipment delivery was scheduled before the consultations, with a pairing and installation guide, which greatly reduced the time of those consultations.

More than 2.8 million records were received by the platform, and the expectation is to reach more than 20 million data records by the end of the project on October 23, 2022.

Reliability studies on data transmission will be carried out as reported in the Methods section, and other studies will also make use of this large mass of data.

As the volunteers were also grouped by disease (COVID-19) status and demographic characteristics, we expect to enable analyses regarding the evolution of COVID-19 and accuracy of the smartwatch with respect to the gold standard equipment, among other possibilities that we are following on a daily basis.

During the follow-up medical appointments during our study, through the digital platform, it was possible to identify a pattern of asymptomatic bradycardia in one of the volunteers, for whom the only symptom was tiredness.

So far, 2 volunteers without previous positive results for COVID-19 were infected by the virus during the study, staying in home isolation and accompanied by the digital platform.

All these data, added to the participants’ individual characteristics and clinical follow-up, will be evaluated and analyzed at the end of the study, thanks to the digital platform that makes the collected data available for continuous monitoring, at a distance, without the need for the intervention of a health professional.

Compared with similar recent studies by Quer et al [9] and Mishra et al [10] that approached the detection of COVID-19 through the use of heart rate, steps, and sleep data, the digital solution in this study also provides researchers and clinicians data about oxygenation during sleep [27], in an organic and continuous way, in addition to data on blood pressure and oxygenation, upon volunteer request.

Furthermore, as very well detailed by Vijayan et al [28], smartwatches have very broad applicability in the health care field. Since the LIKA digital solution was developed together with the manufacturer, it is ready to receive and provide, for clinical use, data from other features not included in this version of the study, as is the case for electrocardiogram for atrial fibrillation monitoring reported in the studies by Nasarre et al [11], Bumgarner et al [12], and Perez et al [13].

For the purpose of clinical use, with no intervention to encourage volunteers to sync the data and automatic synchronization between the digital platform (LIKA-Web) and digital solution on the mobile phone (LIKA-App) set to every 1 hour, the total time delay in synchronization was under 1 hour for less than 0.5% of the records and under 2 hours for only 5% of the records (Table 8).

These results indicate that this solution is not suitable for monitoring critical events, which makes the alert system unsuitable for urgent clinical use.

However, almost 70% of the data are synchronized within 24 hours (Table 8), and less than 10% of the data are synchronized in more than 7 days (Table 8), indicating that the digital solution is more than adequate to be used as a nonurgent health care tool.

Given the LIKA platform was developed with scalability in mind, it is suitable to be used in multicenter, large-scale, and long-term follow-up studies, and it can provide researchers and clinicians with a powerful tool to monitor their patients.

In addition, as the digital solution LIKA is entirely in Brazilian Portuguese and based on cloud technology, it is ready to be accessed in any region of a continental country like Brazil, a region that still lacks studies in this area. Regions in need of medical assistance and located in hard-to-reach areas, such as Amazonas, will be able to take advantage of this technology, as suggested in recent studies carried out by HCFMUSP [29]. Those regions may greatly benefit from a system that enables patient follow-up remotely, such as the one proposed here.

### Limitations

This work is a preliminary report of an ongoing study.

Data are still insufficient for analysis of reliability and accuracy of smartwatches. However, the platform has proven itself ready for data collection on a large scale, and the vital sign values have been used by the medical team in return visits to guide health assessment.

Without in-depth analysis, some records with human error in the data transcription to paper form or to REDCap were identified and confirmed; that is, the data on the digital platform are identical to those recorded on the smartwatches but different from those inputted on the paper form and in REDCap.

Furthermore, this paper reports only part of the project “Predictive monitoring in person-centered care using smartwatches.” Methodologies about the division of the groups of volunteers, clinical follow-up setting, clinical scale used to evaluate COVID-19 patients, and user satisfaction surveys are not part of this preliminary report, as they are not relevant to the operating results of the digital platform,

### Cost

In all, US $39,053.98 (BRL to US $ conversion of 5.80 obtained on December 31, 2021) were spent on smartwatches, gold standard devices, and mobile phones in this project (Table 9).

**Table 9:** Total investment on devices and equipment.

Device	Quantity, n	Investment (US $)
Samsung Galaxy 4 smartwatch	84	19,828.73
Noninvasive blood pressure monitor	92	1866.57
Pulse oximeter for noncontinuous monitoring	92	1130.24
Samsung Galaxy A52 smartphone	45	16,228.44

### Conclusions

The LIKA-Web platform is working correctly in its role of presenting data collected from smartwatches worn by research volunteers on a continuous basis, without the need for human action, to research monitors and clinicians.

Vital sign values ​​are being monitored by the research team as part of monitoring the health of volunteers.

Reliability studies on the digital platform still need to be carried out at the end of the follow-up scheduled for October 23, 2022.

### Future Perspectives

At end of this study, the accuracy and precision of the collected data will be analyzed.

Once it is concluded that the data collected are reliable and have high accuracy, a larger study with volunteers across the country is planned, taking advantage of the structure already built for LIKA-Web.

A new digital solution is planned for development, with which volunteers will not only be able to access their vital signs data but also grant permission to their caregivers, so they can also monitor their health.

In the near future, with the LIKA-Web platform, it will be possible to carry out remote monitoring of an entire community, supporting existing primary care programs and generating alerts of altered vital signs, in order to support health care professionals.

## Abbreviations

cBPM: continuous beats per minute
HCFMUSP: Hospital das Clínicas of The Faculdade de Medicina da Universidade de São Paulo
mHealth: mobile health
REDCap: Research Electronic Data Capture
REM: rapid eye movement
Q1: lower quartile
Q2: median quartile
Q3: upper quartile
